# Prognostic value of inflammatory markers and different treatment regimens in neuroendocrine cervical carcinoma: a retrospective study

**DOI:** 10.3389/fphar.2025.1652092

**Published:** 2025-08-01

**Authors:** Mingqin Kuang, Qi Wang, Ying Yu, Changmei Shen, Tao Peng, Meili Li, Xiyun Cheng, Jing Huang

**Affiliations:** ^1^ Department of Gynecology and Oncology, Ganzhou Cancer Hospital, Ganzhou, China; ^2^ Department of Radiotherapy, Ganzhou Cancer Hospital, Ganzhou, China

**Keywords:** neuroendocrine cervical carcinoma, inflammatory markers, prognosis, clinical research, retrospective study

## Abstract

**Background:**

Neuroendocrine cervical carcinoma (NECC) is a rare and highly aggressive gynecological tumor, with poor prognosis and limited standardized treatment options. Inflammation plays a significant role in tumor progression, and systemic inflammatory markers such as neutrophil-to-lymphocyte ratio (NLR), platelet-to-lymphocyte ratio (PLR), and lymphocyte-to-monocyte ratio (LMR) have shown prognostic value in other malignancies. However, their role in NECC remains unclear.

**Methods:**

This single-center retrospective study included 25 NECC patients treated at our hospital between 2014 and 2024. Patients were divided into three groups based on treatment regimens: paclitaxel plus cisplatin combined with radiotherapy, etoposide plus cisplatin combined with radiotherapy, and radiotherapy alone. Baseline characteristics, inflammatory markers, and clinical outcomes were analyzed. Kaplan-Meier survival analysis and Log-rank tests were used to compare survival differences.

**Results:**

The median survival time was significantly longer in the etoposide plus cisplatin plus radiotherapy group (1,000 days) compared to the paclitaxel plus cisplatin plus radiotherapy group (776 days) and the radiotherapy-alone group (347 days, P = 0.037). The radiotherapy-alone group had significantly higher neutrophil counts (median = 5.46 × 10^9^/L, P = 0.006), platelet counts (median = 282.5 × 10^9^/L, P = 0.017), NLR (median = 4.68, P < 0.05), and PLR (median = 231.93, P < 0.05), while LMR (median = 1.89, P < 0.05) was lower. For postoperative patients, the median survival time was 1,453 days for the surgery plus etoposide plus cisplatin plus radiotherapy group, compared to 987 days for the surgery plus paclitaxel plus cisplatin plus radiotherapy group (P = 0.048).

**Conclusion:**

Combined chemotherapy with etoposide plus cisplatin and radiotherapy significantly improves survival outcomes in NECC patients compared to radiotherapy alone. This regimen may be particularly beneficial for postoperative patients and those with high-risk factors such as lymphovascular space invasion. Further studies are needed to validate these findings and establish standardized treatment protocols for NECC.

## Introduction

Neuroendocrine cervical carcinoma (NECC) is a rare and highly malignant gynecological tumor, accounting for 1.4%–1.5% of all cervical cancers (Lu et al., 2022; Chao et al., 2023; Stolnicu et al., 2020). The biological behavior of NECC differs from that of common cervical squamous cell carcinoma and adenocarcinoma (Inzani et al., 2022; Giordano et al., 2021). It is highly aggressive, with lymph node metastasis and distant metastasis occurring at an early stage (Valenza et al., 2023; Song et al., 2025; Sakurai et al., 2024). The most common histological type of NECC is small-cell neuroendocrine carcinoma (SCNEC), which accounts for 80.4% of NECC cases, followed by large-cell neuroendocrine carcinoma (LCNEC), which accounts for 12.0% (Bellone et al., 2024; Li C. et al., 2024). The prognosis for NECC is extremely poor, with a 5-year survival rate of 30%–46% for early-stage patients and only 0%–15% for late-stage patients (Saraei, Heshmati, and Hosseini, 2024; Kim et al., 2023). Currently, the treatment options for NECC mainly include surgery, chemotherapy, and radiotherapy, but there is a lack of standardized treatment protocols (Jia et al., 2023; Yu et al., 2024; Flores Legarreta et al., 2023; Lyu et al., 2024). Additionally, existing studies have included a limited number of NECC patients, typically as case reports (Wei et al., 2024; Lee and Ji, 2018). This poses significant challenges to clinicians and researchers.

Inflammation plays a crucial role in the occurrence, development, and metastasis of tumors (Boccardi and Marano, 2024; Yuan et al., 2025; Tezcan et al., 2024). Systemic inflammatory markers, such as the neutrophil-to-lymphocyte ratio (NLR), platelet-to-lymphocyte ratio (PLR), and lymphocyte-to-monocyte ratio (LMR), have been extensively studied and have shown prognostic value in lung cancer (Xu et al., 2025; Budin et al., 2024), gastric cancer (Jung et al., 2024; Cui et al., 2024), colorectal cancer (Xun et al., 2024; Pan et al., 2025), bladder cancer (Hu et al., 2025; Chakra et al., 2025), cervical cancer (Sun et al., 2025), and other malignancies (Bizon et al., 2025; Ma et al., 2024; Cocuzza et al., 2024). Small cell cervical carcinoma (SCCC) is a subtype of neuroendocrine cervical carcinoma (NECC). Pan et al. found that the number of CD8^+^ cells at the invasive margin in SCCC patients predicts a favorable clinical outcome. This is associated with an inflammatory phenotype characterized by genes related to the MHC-II complex (CD74), IFN-α/β signaling, and two neuroendocrine subtypes with high expression of ASCL1 or NeuroD1. Among these, the former serves as an independent prognostic factor for overall survival in SCCC (Pan et al., 2024). A meta-analysis that included 15 cohort studies involving 1,336 gastric cancer patients found that high levels of NLR and PLR were associated with poorer overall survival (OS) and progression-free survival (PFS) in gastric cancer patients treated with immune checkpoint inhibitors (ICIs), suggesting a certain link between blood inflammatory indicators and poor tumor prognosis (Tan et al., 2024). Another study based on the National Health and Nutrition Examination Survey (NHANES) dataset analyzed 4,974 cancer survivors to investigate the prognostic value of NLR for all-cause mortality, cardiovascular mortality, and cancer-specific mortality. The study determined that the optimal cutoff value for NLR was 2.61, dividing the subjects into high and low NLR groups. After adjusting for confounding factors such as age, sex, and comorbidities, the results showed a significant association between elevated NLR levels and increased all-cause and cardiovascular mortality. Further analysis revealed that cancer patients with high NLR levels, including those with breast cancer, prostate cancer, non-melanoma skin cancer, colorectal cancer, and melanoma, had higher all-cause and cardiovascular mortality rates compared to those with low NLR levels (He et al., 2024). A study on extensive-stage small-cell lung cancer (SCLC) involving 100 patients aimed to explore the impact of pre-treatment NLR on PFS. The results showed that the median PFS was 6.2 months for the low pre-treatment NLR group and 5.8 months for the high NLR group (p = 0.675), indicating that pre-treatment NLR did not show significant prognostic value in this study (Chen et al., 2024). These differential research conclusions have led us to consider that inflammatory levels may be involved in the progression of malignant tumors, and different inflammatory markers may play different roles in various types of malignancies. These inflammatory markers, by reflecting the body’s inflammatory and immune status, may provide a new perspective for the prognostic assessment of NECC.

Although studies have explored the prognostic value of NLR, PLR, and LMR in other malignant tumors, research on NECC is relatively limited. It is currently unclear whether these inflammatory markers can serve as independent prognostic factors for NECC. Therefore, this study hypothesizes that NLR, PLR, and LMR may be independent prognostic factors for NECC and aims to investigate the impact of different treatment regimens on patient prognosis and assess the relationship between these inflammatory markers and patient outcomes through a retrospective analysis of clinical data from NECC patients diagnosed at our hospital between 2014 and 2024. The significance of this study lies in providing new indicators for prognostic stratification and treatment decision-making in NECC, thereby improving patient treatment outcomes and survival rates.

## Materials and methods

### Study subjects and screening process

**FIGURE 1:**
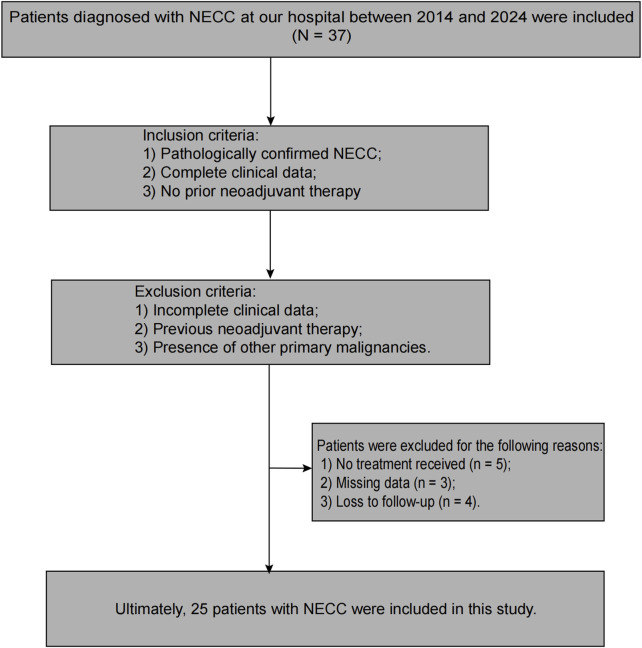
Patient Screening and Enrollment Flowchart. This figure illustrates the process of screening and enrolling patients with NECC from January 2014 to December 2024. A total of 37 patients were initially screened, with 12 excluded due to incomplete data (5 patients who did not receive any treatment after diagnosis, 3 with partial data missing, and 4 with failed follow-up).

This study is a single-center retrospective study that included patients with cervical cancer who were treated at our hospital from January 2014 to December 2024 and were confirmed to have NECC by two associate chief pathologists. The baseline characteristics of the patients included age, FIGO stage, tumor size, tumor volume, deep myometrial invasion (DMI), and lymphovascular space invasion (LVSI). The inclusion criteria were as follows: 1) pathologically confirmed NECC, 2) complete clinical data, and 3) no prior neoadjuvant therapy. The exclusion criteria included: 1) incomplete clinical data, 2) prior neoadjuvant therapy, and 3) concurrent other primary malignancies (Supplementary Table S1). A total of 37 NECC patients were screened, of whom 12 were excluded due to incomplete data. This included five patients who did not receive any treatment after diagnosis, 3 with partial data missing, and 4 with failed follow-up (Figure 1). Patients in the only radiotherapy group were primarily those with advanced-stage tumors or elderly patients who could not tolerate surgery. The paclitaxel - cisplatin - radiotherapy group included patients whose tumors contained partial squamous cell or adenocarcinoma components. The etoposide - cisplatin - radiotherapy group comprised patients with pure neuroendocrine tumors. In this study, radiotherapy was initiated 3–6 weeks after the completion of the last chemotherapy cycle. The exact timing was determined individually, based on each patient’s hematologic parameters and hepatic and renal function, to ensure adequate recovery and tolerance for radiotherapy.

### Definition of NLR, PLR, and LMR

In this study, complete blood counts were performed using an automated hematology analyzer to record the counts of neutrophils, lymphocytes, platelets, and monocytes. The time point of testing was at the initial hospital admission and before treatment. Subsequently, the NLR, PLR, and LMR were calculated. The formulas for calculating NLR, PLR, and LMR are as follows: 1) NLR = neutrophil count/lymphocyte count, 2) PLR = platelet count/lymphocyte count, and 3) LMR = lymphocyte count/monocyte count.

### Postoperative radiotherapy procedure for NECC

**FIGURE 2:**
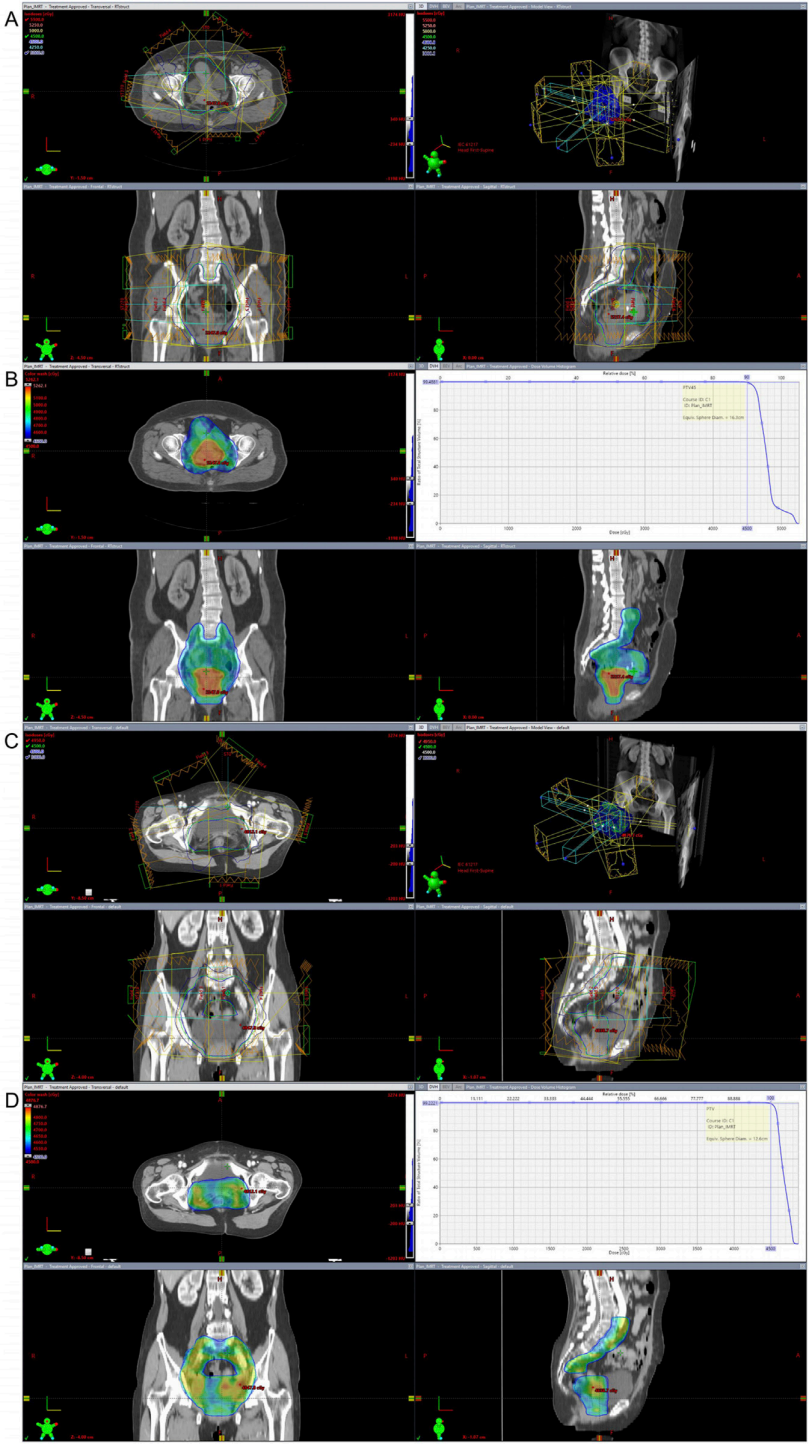
Radiotherapy Field and Dose Distribution Maps. **(A,B)** The tumor coverage for partially NECC patients receiving a radiotherapy dose of 45Gy, with coverage reaching 99.4881%. **(C,D)** The similar coverage of 99.2221% for the other patients. These results demonstrate the high accuracy of the radiotherapy treatment range.

Patients underwent a comprehensive evaluation to determine the suitability for radiotherapy, integrating surgical records, pathology reports, and imaging data. Radiotherapy was delivered with a total dose range of 45 Gy, administered in 10–33 fractions. Treatment planning was conducted by experienced radiation oncologists, who delineated the tumor target area and critical normal tissues using CT simulation. The radiotherapy technique utilized was either intensity-modulated radiation therapy (IMRT), optimized to maximize tumor coverage while minimizing exposure to normal tissues. Position and dose verification were performed using cone beam computed tomography (CBCT), with initial and regular checks throughout the treatment course. Follow-up assessments were conducted to evaluate therapeutic effects and manage adverse reactions. From the radiotherapy field and dose distribution maps of some patients with cervical neuroendocrine carcinoma, it can be seen that when the radiotherapy dose was 45Gy, the tumor coverage for some patients was 99.4881% (Figures 2A,B). Similarly, from the radiotherapy field and dose distribution maps of other patients with cervical neuroendocrine carcinoma, it can be observed that when the radiotherapy dose was 45Gy, the tumor coverage was 99.2221% (Figures 2C,D). This indicates that, from a technical standpoint, the treatment range was highly accurate, with minimal error between the two groups of patients, which was not statistically significant.

### Follow-up methods

During the treatment course, follow-up was conducted monthly, with timely reminders for patients to be hospitalized and to review their complete blood counts and liver and kidney function results. After the completion of the treatment course, the follow-up frequency was changed to once every 3 months. Telephone follow-up mainly involved inquiries, while outpatient follow-up required further detailed medical history consultation and examinations, including symptom assessment, physical examination, and pelvic examination. The endpoint events were defined as death, tumor recurrence, or progression. The follow-up cutoff date was 31 December 2024. During the follow-up period, all patients were contacted either by phone or through outpatient visits, with no loss to follow-up.

### Statistical analysis

The analysis software used in this study were R (version 4.4.1) and Empower Stats (X&Y Solutions Inc., Boston, MA; available at www.empowerstats.com). The specific methods included: 1) Descriptive statistics: continuous variables were expressed as medians and interquartile ranges, while categorical variables were presented as frequencies and percentages. 2) Group comparisons: t-tests were used for continuous variables, and chi-square tests were used for categorical variables. 3) Survival analysis: Kaplan-Meier method was employed to plot survival curves, and the Log-rank test was used to compare survival differences between groups.

## Results

### Grouping of NECC patients, baseline characteristics, and correlation with treatment efficacy

This study included 25 NECC patients, divided into three groups based on treatment regimens: paclitaxel plus cisplatin combined with radiotherapy, etoposide plus cisplatin combined with radiotherapy, and radiotherapy alone. Baseline analysis revealed that the median age of patients in the radiotherapy-alone group was significantly higher than that in the combined chemotherapy groups (P = 0.046), which may be related to the poorer tolerance of chemotherapy in elderly patients. Survival analysis indicated that the median survival time in the etoposide plus cisplatin plus radiotherapy group was 1,000 days, significantly longer than that in the paclitaxel plus cisplatin plus radiotherapy group (776 days) and the radiotherapy-alone group (347 days, P = 0.037), suggesting that combined chemotherapy can improve survival prognosis. In terms of hematological indicators, the radiotherapy-alone group had significantly higher neutrophil counts (median 5.46 × 10^9^/L, P = 0.006), platelet counts (median 282.5 × 10^9^/L, P = 0.017), NLR (median 4.68, P < 0.05), and PLR (median 231.93, P < 0.05), while LMR (median 1.89, P < 0.05) was significantly lower compared to the combined chemotherapy groups, indicating that patients receiving radiotherapy alone may have more pronounced inflammatory reactions and immune suppression (Table 1).

**TABLE 1:** Population baseline under three different treatment regimens.

Group	Paclitaxel + Cisplatin + Radiotherapy	Etoposide + Cisplatin + Radiotherapy	Only radiotherapy	P-value
N	10	10	4	
Age, years	48.00 (44.75–50.50)	53.00 (44.50–58.25)	64.50 (54.75–74.00)	0.046
HT, cm	154.00 (151.00–158.00)	150.00 (146.25–151.75)	150.50 (147.25–154.00)	0.121
WT, cm	59.00 (51.50–64.00)	48.00 (43.00–54.25)	53.00 (51.50–55.50)	0.103
BMI, kg/m^2^	23.94 (22.37–26.51)	22.23 (19.66–23.32)	24.06 (23.39–24.42)	0.214
Tumor volume, cm^3^	8.83 (1.84–24.45)	12.04 (4.81–26.25)	10.16 (3.83–19.15)	0.839
Maximum tumor diameter, mm	35.00 (18.50–49.50)	34.00 (27.00–44.00)	30.00 (21.75–40.50)	0.984
survival time, day	776.00 (555.25–937.50)	1000.00 (482.50–1423.50)	347.00 (308.50–410.75)	0.037
Neutrophil count, 10^9/L	3.67 (2.87–4.81)	5.68 (4.75–6.55)	5.46 (4.90–5.73)	0.006
platelet count, 10^9/L	202.00 (164.75–240.75)	267.00 (229.25–307.00)	282.50 (240.75–315.00)	0.017
Mononuclear cell count, 10^9/L	0.56 (0.43–0.68)	0.33 (0.22–0.45)	0.61 (0.30–1.01)	0.044
Lymphocyte count, 10^9/L	2.66 (2.14–3.17)	2.25 (1.64–2.68)	1.17 (1.00–1.31)	0.004
NLR	1.28 (1.02–2.14)	2.42 (2.06–3.47)	4.68 (3.82–6.06)	0.002
PLR	74.80 (56.37–105.41)	127.36 (105.62–164.05)	231.93 (205.56–290.39)	0.001
LMR	4.33 (3.96–5.16)	6.89 (5.73–8.32)	1.89 (1.54–2.84)	0.016
Hemoglobin, g/L	120.50 (110.50–132.75)	126.00 (106.75–138.75)	111.00 (95.75–126.25)	0.649
Uric acid, mg/dL	275.65 (249.22–341.43)	262.75 (211.00–283.05)	379.85 (302.27–462.02)	0.264
Albumin, g/L	43.05 (40.65–44.48)	43.00 (41.40–46.27)	44.25 (42.42–45.70)	0.720
HDL, mg/dL	1.18 (1.17–1.20)	1.11 (1.06–1.13)	1.02 (1.00–1.12)	0.221
TG, mg/dL	1.71 (1.12–2.04)	1.48 (0.89–2.98)	3.00 (2.42–3.46)	0.151
SCCA, μg/L	1.21 (0.67–3.59)	1.17 (0.62–1.70)	6.36 (1.95–11.03)	0.116
HPV, %				0.604
No	2 (20.00)	4 (40.00)	1 (25.00)	
Yes	8 (80.00)	6 (60.00)	3 (75.00)	
Stage, %				0.075
I	5 (50.00)	5 (50.00)	0 (0.00)	
II	2 (20.00)	0 (0.00)	3 (75.00)	
III	1 (10.00)	2 (20.00)	1 (25.00)	
IV	2 (20.00)	3 (30.00)	0 (0.00)	
DMI, %				0.301
No	3 (30.00)	6 (60.00)	1 (25.00)	
Yes	7 (70.00)	4 (40.00)	3 (75.00)	
LVSI, %				0.018
No	2 (20.00)	6 (60.00)	4 (100.00)	
Yes	8 (80.00)	4 (40.00)	0 (0.00)	

Given the significantly better prognosis in the chemotherapy groups compared to the radiotherapy-alone group, this study further compared the baseline characteristics of the two combined chemotherapy regimens. No significant differences were found between the paclitaxel plus cisplatin group and the etoposide plus cisplatin group in terms of age, tumor volume, or stage (P > 0.05). However, significant differences were observed in height (median 150.00 cm vs 158.00 cm, P = 0.049), weight (median 48.00 kg vs 55.00 kg, P = 0.050), BMI (median 22.23 kg/m^2^ vs 24.15 kg/m^2^, P = 0.109), and hematological indicators. Specifically, the etoposide plus cisplatin group had higher neutrophil counts (median 5.68 × 10^9^/L, P = 0.003), platelet counts (median 267.00 × 10^9^/L, P = 0.006), NLR (median 2.42, P = 0.007), and PLR (median 127.36, P = 0.006), while monocyte counts (median 0.33 × 10^9^/L, P = 0.009) and LMR (median 6.89, P = 0.045) were lower, suggesting that the two regimens have differential effects on immune and inflammatory status (Table 2).

**TABLE 2:** Baseline populations under two different chemotherapy regimens.

Group	Paclitaxel + Cisplatin + Radiotherapy	Etoposide + Cisplatin + Radiotherapy	P-value
N	10	10	
Age, years	48.00 (44.75–50.50)	53.00 (44.50–58.25)	0.294
HT, cm	154.00 (151.00–158.00)	150.00 (146.25–151.75)	0.049
WT, cm	59.00 (51.50–64.00)	48.00 (43.00–54.25)	0.050
BMI, kg/m^2^	23.94 (22.37–26.51)	22.23 (19.66–23.32)	0.109
Tumor volume, cm^3^	8.83 (1.84–24.45)	12.04 (4.81–26.25)	0.622
Maximum tumor diameter, mm	35.00 (18.50–49.50)	34.00 (27.00–44.00)	0.762
survival time, day	776.00 (555.25–937.50)	1000.00 (482.50–1423.50)	0.209
Neutrophil count, 10^9/L	3.67 (2.87–4.81)	5.68 (4.75–6.55)	0.003
platelet count, 10^9/L	202.00 (164.75–240.75)	267.00 (229.25–307.00)	0.006
Mononuclear cell count, 10^9/L	0.56 (0.43–0.68)	0.33 (0.22–0.45)	0.009
Lymphocyte count, 10^9/L	2.66 (2.14–3.17)	2.25 (1.64–2.68)	0.112
NLR	1.28 (1.02–2.14)	2.42 (2.06–3.47)	0.007
PLR	74.80 (56.37–105.41)	127.36 (105.62–164.05)	0.006
LMR	4.33 (3.96–5.16)	6.89 (5.73–8.32)	0.045
Hemoglobin, g/L	120.50 (110.50–132.75)	126.00 (106.75–138.75)	0.650
Uric acid, mg/dL	275.65 (249.22–341.43)	262.75 (211.00–283.05)	0.435
Albumin, g/L	43.05 (40.65–44.48)	43.00 (41.40–46.27)	0.599
HDL, mg/dL	1.18 (1.17–1.20)	1.11 (1.06–1.13)	0.063
TG, mg/dL	1.71 (1.12–2.04)	1.48 (0.89–2.98)	0.459
SCCA, μg/L	1.21 (0.67–3.59)	1.17 (0.62–1.70)	0.157
HPV, %			0.329
No	2 (20.00)	4 (40.00)	
Yes	8 (80.00)	6 (60.00)	
Stage, %			0.469
I	5 (50.00)	5 (50.00)	
II	2 (20.00)	0 (0.00)	
III	1 (10.00)	2 (20.00)	
IV	2 (20.00)	3 (30.00)	
DMI, %			0.178
No	3 (30.00)	6 (60.00)	
Yes	7 (70.00)	4 (40.00)	
LVSI, %			0.068
No	2 (20.00)	6 (60.00)	
Yes	8 (80.00)	4 (40.00)	

Surgery is one of the key factors determining the prognosis of NECC patients. Therefore, we further screened the subjects included in the different chemotherapy regimens. After excluding two patients in the paclitaxel group and five non-surgical patients in the etoposide group, the results showed that the median survival time for the surgery plus etoposide plus cisplatin plus radiotherapy group (n = 5) was 1,453 days, significantly longer than that for the surgery plus paclitaxel plus cisplatin plus radiotherapy group (n = 8, median survival time 987 days, P = 0.048). Hematological indicators revealed that the former group had higher neutrophil counts (median 5.36 × 10^9^/L, P = 0.006), platelet counts (median 276.00 × 10^9^/L, P = 0.015), and PLR (median 140.74, P = 0.003), while monocyte counts (median 0.26 × 10^9^/L, P = 0.007), lymphocyte counts (median 1.81 × 10^9^/L, P = 0.046), and NLR (median 2.95, P = 0.011) were lower, suggesting that surgery combined with etoposide plus cisplatin plus radiotherapy may be more beneficial for long-term survival and immune regulation in NECC patients (Table 3).

**TABLE 3:** Baseline populations under two different chemotherapy regimens after surgery.

Group	Surgery + Paclitaxel + Cisplatin + Radiotherapy	Surgery + Etoposide + Cisplatin + Radiotherapy	P-value
N	8	5	
Age, years	47.00 (44.00–49.75)	52.00 (49.00–56.00)	0.303
HT, cm	154.00 (150.25–156.00)	150.00 (147.00–151.00)	0.091
WT, cm	59.00 (52.50–64.00)	52.00 (44.00–55.00)	0.156
BMI, kg/m^2^	23.94 (22.37–26.24)	23.50 (21.94–24.06)	0.557
Tumor volume, cm^3^	8.83 (2.48–23.86)	2.96 (0.59–10.37)	0.242
Maximum tumor diameter, mm	35.00 (18.75–48.50)	25.00 (15.00–33.00)	0.261
Survival time, day	736.00 (603.75–985.50)	1453.00 (988.00–1530.00)	0.048
Duration of hospitalization, day	17.00 (13.25–22.00)	15.00 (15.00–21.00)	0.259
Neutrophil count, 10^9/L	3.29 (2.67–3.98)	5.36 (4.67–6.59)	0.006
platelet count, 10^9/L	184.50 (154.50–219.50)	276.00 (242.00–295.00)	0.015
Mononuclear cell count, 10^9/L	0.50 (0.41–0.62)	0.26 (0.16–0.31)	0.007
Lymphocyte count, 10^9/L	2.66 (2.02–3.12)	1.81 (1.35–2.04)	0.046
NLR	1.18 (0.96–1.54)	2.95 (2.29–3.64)	0.011
PLR	68.56 (55.12–89.56)	140.74 (135.29–171.82)	0.003
LMR	4.63 (4.19–6.42)	7.97 (7.85–8.44)	0.143
Hemoglobin, g/L	120.50 (109.25–134.50)	135.00 (124.00–140.00)	0.169
Uric acid, mg/dL	275.65 (251.68–308.27)	274.20 (261.00–286.00)	0.770
Albumin, g/L	42.05 (40.15–44.42)	47.00 (42.00–48.00)	0.261
HDL, mg/dL	1.18 (1.17–1.23)	1.11 (1.04–1.11)	0.186
TG, mg/dL	1.71 (1.08–2.04)	2.17 (1.29–4.25)	0.158
SCCA, μg/L	1.21 (0.60–6.17)	0.89 (0.58–1.72)	0.264
HPV, %			0.569
No	2 (25.00)	2 (40.00)	
Yes	6 (75.00)	3 (60.00)	
Stage, %			0.401
I	4 (50.00)	3 (60.00)	
II	2 (25.00)	0 (0.00)	
III	1 (12.50)	2 (40.00)	
IV	1 (12.50)	0 (0.00)	
DMI, %			0.429
No	3 (37.50)	3 (60.00)	
Yes	5 (62.50)	2 (40.00)	
LVSI, %			0.188
No	0 (0.00)	1 (20.00)	
Yes	8 (100.00)	4 (80.00)	

### Correlation Heatmap analysis of blood indicators and inflammatory markers

**FIGURE 3:**
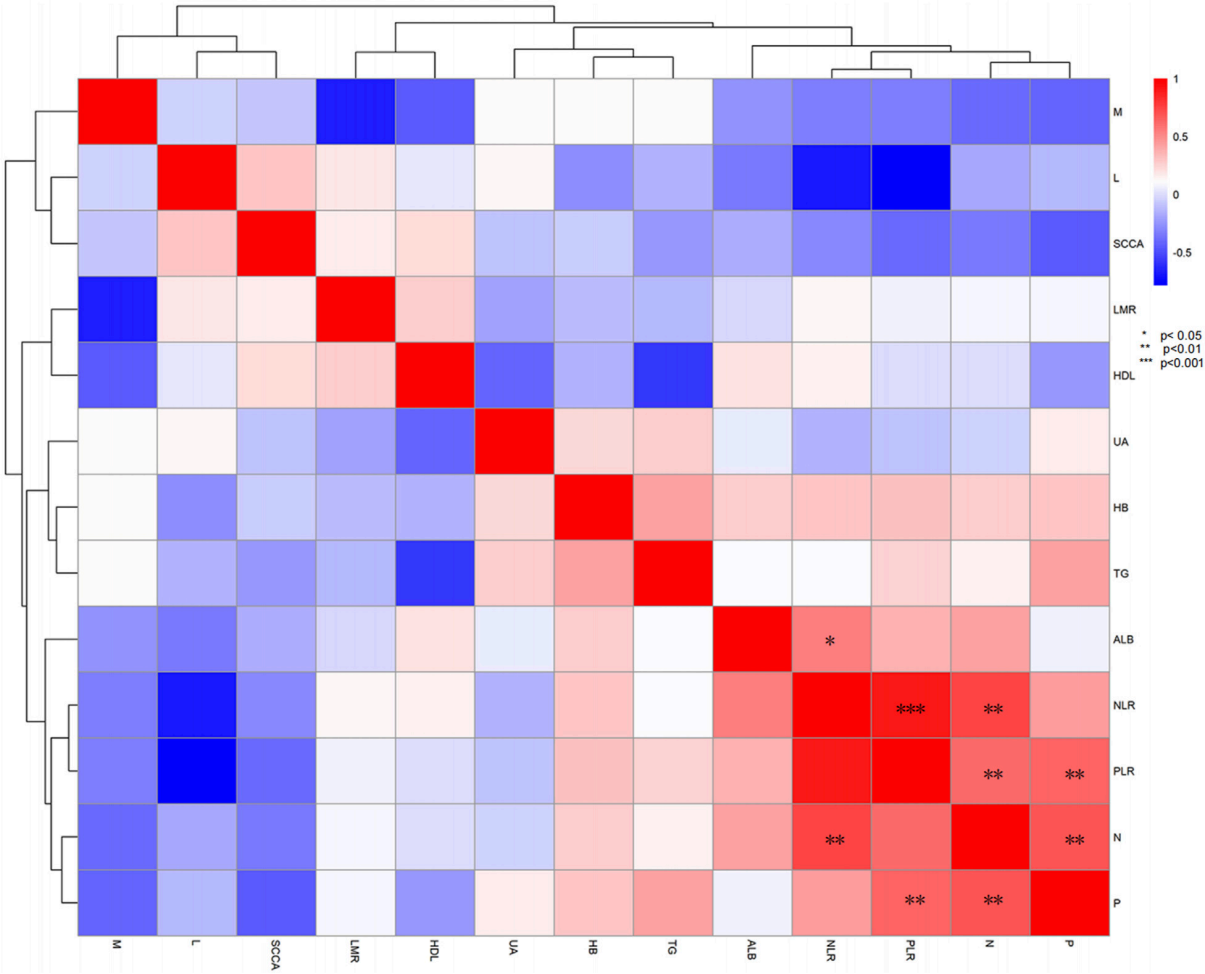
Correlation Heatmap of Blood Indicators and Inflammatory Markers. This heatmap displays the correlations between SCCA, NLR, LMR, and ALB. LMR is significantly negatively correlated with NLR. Albumin shows a moderate negative correlation with NLR. * indicates p < 0.05, ** indicates p < 0.01, and *** indicates p < 0.001.

This study used heatmap analysis to reveal the correlations between blood indicators and inflammatory markers. The LMR was significantly negatively correlated with NLR, consistent with the inverse relationship defined by their ratios, confirming the regulatory role of inflammatory status on immune cell distribution. Albumin (ALB) showed a moderate negative correlation with NLR, indicating that patients with good nutritional status may have lower inflammatory loads. These correlations provide clues for exploring the role of biomarkers in the diagnosis, prognostic assessment, and treatment response of NECC, suggesting that combined monitoring of SCCA, NLR, LMR, and ALB levels may offer reference for individualized treatment planning (Figure 3).

### Clinical and pathological characteristics of NECC patients with different treatment regimens

**FIGURE 4:**
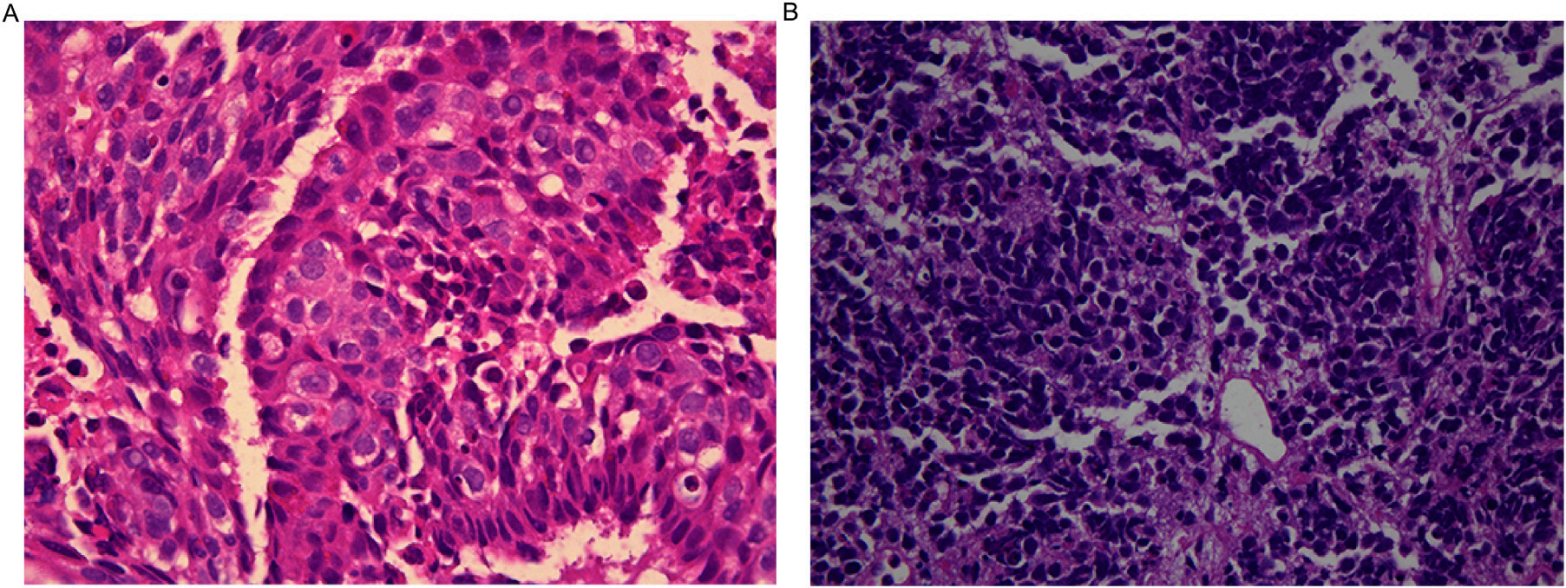
Histological Subtypes of NECC Patients. **(A)** 13 patients with cervical neuroendocrine carcinoma combined with partial squamous cell carcinoma, adenocarcinoma, or adenosquamous carcinoma. **(B)** 22 patients with pure cervical neuroendocrine carcinoma.

**FIGURE 5:**
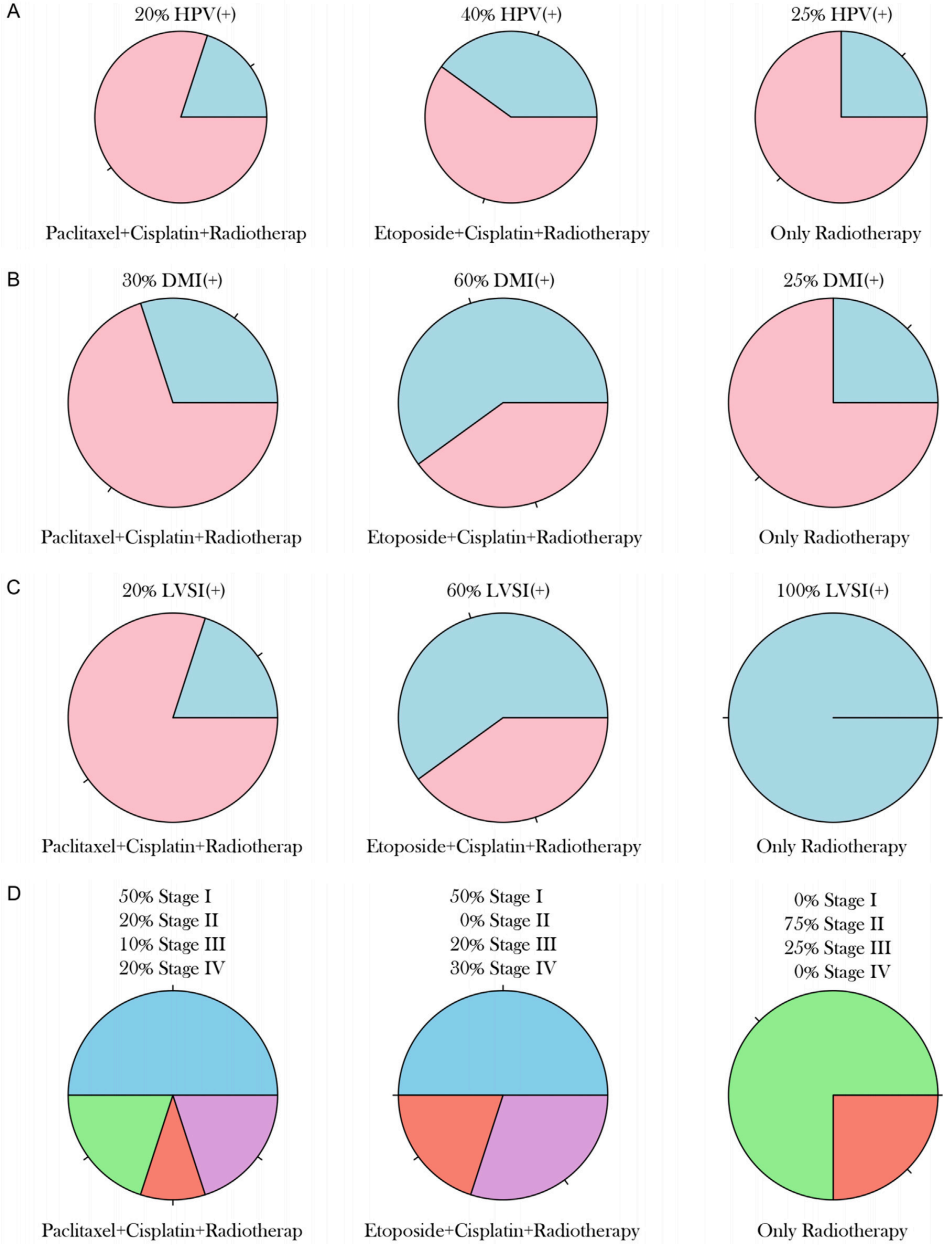
Clinical and Pathological Characteristics by Treatment Group. **(A)** HPV positivity rates (40% in the etoposide plus cisplatin plus radiotherapy group, 20% in the paclitaxel plus cisplatin plus radiotherapy group, and 25% in the radiotherapy-alone group, P = 0.042). **(B)** DMI rates (60% in the etoposide group, 25% in the paclitaxel group, and 30% in the radiotherapy-alone group, P = 0.038). **(C)** LVSI rates (100% in the radiotherapy-alone group, 20% in the paclitaxel group, and 60% in the etoposide group, P = 0.018). **(D)** Tumor stage distribution (50% Stage I in both chemotherapy groups, 75% Stage II in the radiotherapy-alone group, P = 0.075).

Among the 25 NECC patients included, 13 had cervical neuroendocrine carcinoma with partial squamous cell carcinoma, adenocarcinoma, or adenosquamous carcinoma (Figure 4A), while 22 had pure cervical neuroendocrine carcinoma (Figure 4B). Further analysis of the clinical and pathological characteristics of NECC patients with different treatment regimens showed significant differences among the three groups in terms of HPV infection status, DMI, LVSI, and tumor stage. The highest HPV positivity rate was observed in the etoposide plus cisplatin plus radiotherapy group (40%), while the lowest was in the paclitaxel plus cisplatin plus radiotherapy group (20%), with the radiotherapy-alone group at 25% (Figure 5A, P = 0.042). The DMI positivity rate was highest in the etoposide plus cisplatin plus radiotherapy group (60%), followed by the radiotherapy-alone group and the paclitaxel plus cisplatin plus radiotherapy group at 25% and 30%, respectively (Figure 5B, P = 0.038). The LVSI positivity rate reached 100% in the radiotherapy-alone group, significantly higher than in the paclitaxel plus cisplatin plus radiotherapy group (20%) and the etoposide plus cisplatin plus radiotherapy group (60%, Figure 5C, P = 0.018), suggesting that combined chemotherapy may have a positive effect on inhibiting lymphovascular invasion. The tumor stage distribution showed that 50% of patients in both the paclitaxel plus cisplatin plus radiotherapy group and the etoposide plus cisplatin plus radiotherapy group were in Stage I, while 75% of patients in the radiotherapy-alone group were in Stage II (Figure 5D, P = 0.075). The proportion of Stage II patients was significantly higher in the radiotherapy-alone group (75%), while the combined chemotherapy groups mainly consisted of patients in Stage I (50%) and Stage IV (20%), with this difference being related to the preference for treatment regimen selection in clinical practice. That is, patients with advanced disease are more likely to receive combined chemotherapy to enhance therapeutic efficacy. These results suggest that the distribution of clinical and pathological characteristics may influence the choice of treatment regimen and prognosis, and combined chemotherapy may be more clinically valuable for patients with high-risk factors such as LVSI.

### Surgery combined with etoposide plus cisplatin plus radiotherapy May Be the optimal treatment regimen for NECC patients

**FIGURE 6:**
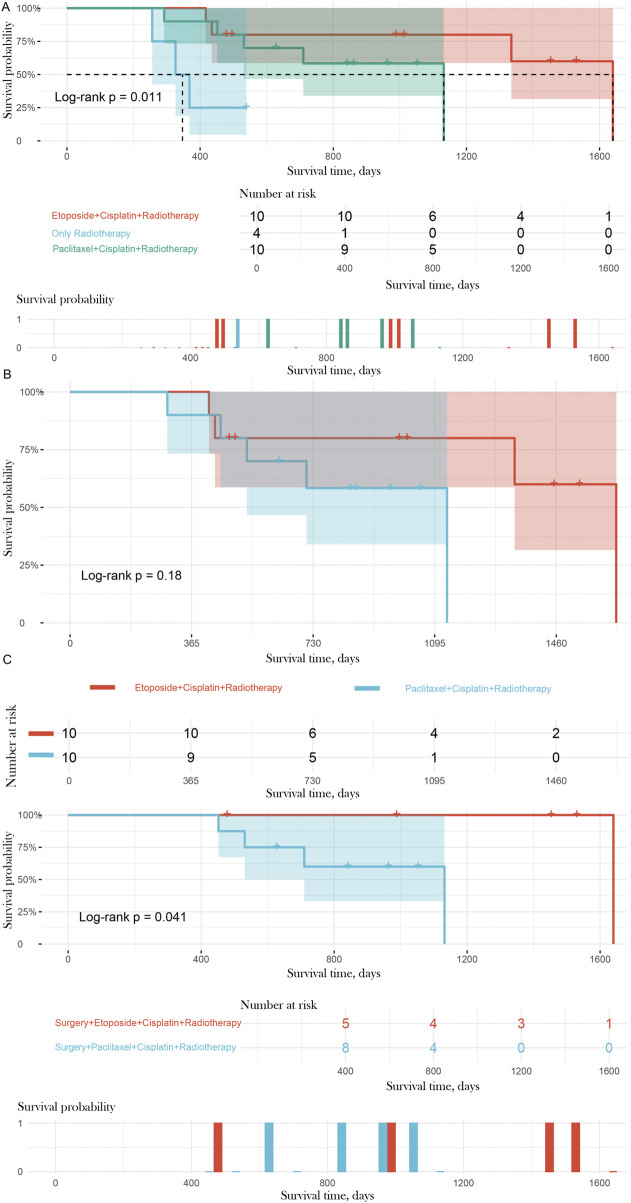
Survival Curves by Treatment Regimen. **(A)** Patients receiving combined chemotherapy (etoposide plus cisplatin plus radiotherapy and paclitaxel plus cisplatin plus radiotherapy) had significantly better survival compared to those receiving radiotherapy alone (P = 0.011). **(B)** Compares the survival curves of the two chemotherapy regimens, showing no significant difference (P = 0.18). **(C)** Postoperative patients receiving surgery plus etoposide plus cisplatin plus radiotherapy had significantly better survival compared to those receiving surgery plus paclitaxel plus cisplatin plus radiotherapy (P = 0.041).

Survival curve analysis showed that the survival time of patients in the etoposide plus cisplatin plus radiotherapy group and the paclitaxel plus cisplatin plus radiotherapy group was significantly better than that in the radiotherapy-alone group (Figure 6A, P = 0.011), indicating that combined chemotherapy significantly improves the prognosis of NECC patients compared to radiotherapy alone. After excluding the radiotherapy-alone group, the survival curves of the two combined chemotherapy regimens showed similar downward trends (Figure 6B, P = 0.18), with no significant difference, suggesting that the two chemotherapy regimens have no significant difference in their impact on survival when surgery is not considered.

Further stratified analysis of postoperative patients revealed that the survival curve of the surgery plus etoposide plus cisplatin plus radiotherapy group was significantly better than that of the surgery plus paclitaxel plus cisplatin plus radiotherapy group (Figure 6C, P = 0.041). This result suggests that for postoperative NECC patients, the combination of etoposide with cisplatin and radiotherapy is more beneficial for prolonging survival time compared to the paclitaxel regimen. Combined with the previous analysis of clinical and pathological characteristics, surgery combined with etoposide plus cisplatin plus radiotherapy may offer superior prognostic value for NECC patients with high-risk factors, providing important reference for the selection of individualized treatment regimens in NECC patients.

## Discussion

NECC is a rare and highly aggressive gynecological malignancy (Sun et al., 2022; Wang et al., 2024; Paterniti et al., 2021). Due to its low sensitivity to traditional treatments and extremely poor prognosis, it remains a significant challenge in clinical diagnosis and treatment (He et al., 2025; Kuji et al., 2023). Numerous studies have shown that systemic inflammation plays a key role in the development and progression of tumors (Hou et al., 2021; Nøst et al., 2021; Korbecki et al., 2021). Among them, hematological indicators such as the NLR, PLR, and LMR have shown unique advantages in tumor prognosis assessment because they are easy to obtain and can effectively reflect the systemic inflammatory state (Zinellu et al., 2022; Martinez-García et al., 2022; Di Rosa et al., 2023). However, clinical research on NLR, PLR, and LMR in NECC patients is still a blank. This study aims to systematically analyze the impact of different treatment regimens on the prognosis of NECC patients and to explore the potential prognostic value of systemic inflammatory markers in this disease, providing a basis for optimizing clinical treatment strategies.

The stratified analysis of postoperative patients in this study showed that the regimen combining surgery with etoposide and cisplatin as well as radiotherapy was associated with the longest median survival time (1,453 days), while the median survival time for the group with surgery combined with paclitaxel, cisplatin, and radiotherapy was 987 days (P = 0.048). Previous studies have explored the pathological characteristics of NECC (Wang et al., 2025; Ordulu et al., 2022). For example, Sagnic et al. retrospectively analyzed 9 cases of NECC diagnosed among 453 cervical cancer patients at the Akniz University Gynecological Oncology Outpatient Clinic from 2004 to 2021 and found that the average overall survival of this cohort was 26 months, with a 5-year survival rate of 53.3% and a progression-free survival of 62.5%. The study proposed that surgery combined with adjuvant chemoradiotherapy is the optimal treatment regimen, with cisplatin/carboplatin combined with etoposide being the most commonly used chemotherapy regimen (Sağnıç et al., 2021). This conclusion is similar to our study, but our study further confirmed that surgery combined with cisplatin + etoposide regimen may be an optimized treatment strategy for NECC patients through a larger sample size of systematic analysis and purposeful group comparison, providing more convincing evidence-based basis for clinical decision-making in this field.

In an international multicenter study that included 10,575 cases of invasive cervical cancer (ICC), 49 cases with neuroendocrine features were identified through histological examination. Subsequently, 13 samples were further screened for HPV DNA testing using the SPF10/DEIA/LIPA25 system, and the results showed that the HPV16 infection rate in NECC patients was 54.8%, and HPV18 was 40.5% (Alejo et al., 2018). The study pointed out that the existing HPV vaccines can largely prevent the occurrence of such aggressive tumors, providing a theoretical basis for including HPV infection status in the baseline analysis of this study. It is worth noting that no statistical difference in HPV infection rates was found among the three groups of patients in this study (P > 0.05), which may be related to the limited sample size. Further research is needed to explore the potential association between HPV infection load and the clinical characteristics and treatment response of NECC by expanding the sample size.

Inflammation, as a key component of the tumor microenvironment, plays an indispensable role in the occurrence, development, and outcome of cervical cancer (Ma et al., 2025; Pandey et al., 2024). Many cutting-edge studies have thoroughly analyzed the intrinsic connection between inflammation-related indicators and cervical cancer from multiple dimensions, including molecular mechanisms, prognostic assessment, and diagnostic biomarkers (Zhou et al., 2021; Zhao et al., 2024; Markus, 2024; Li Q. et al., 2024). In terms of molecular mechanisms, some studies have used high-throughput sequencing technology to systematically compare the differential expression profiles of microRNAs in cervical cancer tissues and healthy tissues, and predicted potential regulatory networks through bioinformatics analysis. Subsequent functional experiments such as cell transfection and luciferase reporter genes have confirmed that miR-30a and miR-34c can target key proteins in the JAK1/STAT3 signaling pathway, thereby participating in the regulation of inflammation induced by HPV. This finding reveals the molecular mechanism by which HPV virus activates the expression of inflammation-related genes through miRNA-mediated signaling pathways, ultimately promoting the malignant transformation of cervical cells (Yan et al., 2024). In the field of prognostic assessment, a retrospective study conducted by Li et al. included 212 patients with locally advanced cervical cancer (LACC) who received concurrent chemoradiotherapy (CCRT). By constructing a Cox proportional hazards model, the study systematically evaluated the correlation between the systemic immune-inflammation index (SII) and patient survival outcomes. The results showed that after adjusting for confounding factors such as age, tumor stage, and treatment regimen, SII can still serve as an independent prognostic factor. Patients with higher SII values have significantly lower 5-year overall survival rates and progression-free survival rates (Ntuli et al., 2022). In the exploration of diagnostic biomarkers, Toth’s team conducted a comprehensive analysis of 395 patients who underwent loop electrosurgical excision procedure (LEEP). The study found that the NLR is not only significantly associated with p16 protein positivity (p = 0.011) and HPV DNA positivity (p = 0.04), but also the mean NLR value of HPV-positive patients is 33.5% higher than that of negative patients. Through ROC curve analysis, the AUC for NLR to diagnose HPV infection reached 0.610, showing moderate diagnostic efficacy. This indicates that NLR may serve as a low-cost and easily obtainable auxiliary indicator for screening HPV-related cervical lesions (Tóth et al., 2025). However, our study focuses on the interaction between inflammation and tumor markers and nutritional status, and found that SCCA is positively correlated with NLR, which means that intensified inflammatory response may synergistically promote tumor progression. At the same time, the LMR and serum albumin levels are negatively correlated with NLR, indicating that the inflammatory state may deplete the body’s immune and nutritional reserves. These findings collectively suggest that combined monitoring of SCCA, NLR, LMR, and albumin levels can provide a more comprehensive disease assessment for cervical cancer patients from multiple dimensions, including inflammatory response, tumor burden, immune function, and nutritional status. It is expected to become an important strategy for guiding the formulation of individualized treatment plans and dynamically monitoring treatment effects, providing a new research direction and practical basis for improving the diagnosis and treatment level of cervical cancer.

There are some limitations in this study that need to be acknowledged. First, the retrospective design of the study and the relatively small sample size limit the generalizability of the study results. Second, the lack of standardized treatment protocols for NECC across different centers may introduce bias in the comparison of treatment outcomes. Future research needs to conduct larger prospective studies with standardized treatment protocols to verify these findings and develop clearer diagnostic and treatment plans for the management of NECC. However, despite the above shortcomings, this study emphasizes the potential benefits of combining etoposide and cisplatin chemotherapy regimen with radiotherapy in improving the survival prognosis of NECC patients. The study results also show that systemic inflammatory markers may serve as useful prognostic indicators for this patient population. Further research is needed in the future to explore their potential mechanisms and develop more effective treatment strategies for this rare and aggressive malignancy.

## Conclusion

In this study, we retrospectively analyzed 25 patients with NECC and found that systemic inflammatory markers, particularly NLR, PLR, and LMR, measured at initial hospital admission were significantly associated with treatment response and prognosis. Patients treated with combined chemotherapy had better survival outcomes than those who received radiotherapy alone. Among them, the etoposide-based regimen, especially when combined with surgery, showed the most favorable prognosis. These findings suggest that pre-treatment inflammatory markers may serve as independent prognostic indicators and that individualized treatment strategies based on both tumor characteristics and systemic immune status could improve clinical outcomes in NECC patients.

## Data Availability

The raw data supporting the conclusions of this article will be made available by the authors, without undue reservation.

## Abbreviations

NECC, Neuroendocrine cervical carcinoma; SCNEC, small-cell neuroendocrine carcinoma; LCNEC, large-cell neuroendocrine carcinoma; NLR, neutrophil-to-lymphocyte ratio; PLR, platelet-to-lymphocyte ratio; LMR, lymphocyte-to-monocyte ratio; OS, overall survival; ICIs, immune checkpoint inhibitors; NHANES, the National Health and Nutrition Examination Survey; SCLC, small-cell lung cancer; DMI, deep myometrial invasion; LVSI, lymphovascular space invasion; IMRT, intensity-modulated radiation therapy; CBCT, cone beam computed tomography; LACC, locally advanced cervical cancer; CCRT, concurrent chemoradiotherapy.
