# Supplementary material for: Prognostic value of inflammatory markers and different treatment regimens in neuroendocrine cervical carcinoma: a retrospective study
Source: Front Pharmacol. 2025 Aug 1;16:1652092. doi: 10.3389/fphar.2025.1652092 (PMC12354512; doi:10.3389/fphar.2025.1652092)
Supplement: Supplementary file 1 [file Supplementaryfile1.docx]

Supplementary Table 1: Inclusion and exclusion criteria of this study.

| Inclusion criteria included: | Exclusion criteria included: |
| --- | --- |
| 1) pathologically confirmed NECC | 1) incomplete clinical data |
| 2) complete clinical data | 2) prior neoadjuvant therapy |
| 3) no prior neoadjuvant therapy. | 3) concurrent other primary malignancies |
